# Antioxidant and antiproliferative effect of a glycosaminoglycan extract from *Rapana venosa* marine snail

**DOI:** 10.1371/journal.pone.0297803

**Published:** 2024-02-15

**Authors:** Alexandra Gaspar-Pintiliescu, Laura M. Stefan, Elena Mihai, Catalina Sanda, Vasile S. Manoiu, Daniela Berger, Oana Craciunescu

**Affiliations:** 1 Department of Cellular and Molecular Biology, National Institute of Research and Development for Biological Sciences, Bucharest, Romania; 2 Faculty of Chemical Engineering and Biotechnologies, University "Politehnica" of Bucharest, Bucharest, Romania; CRCL: Centre de Recherche en Cancerologie de Lyon, FRANCE

## Abstract

Marine glycosaminoglycans (GAG) isolated from different invertebrates, such as molluscs, starfish or jellyfish, have been described as unique molecules with important pharmacological applications. Scarce information is available on GAG extract from *Rapana venosa* marine snail. The aim of this study was to isolate a GAG extract from *R*. *venosa* marine snail and to investigate its physicochemical, antioxidant and antiproliferative properties for further biomedical use. The morphology, chemical and elemental composition of the extract were established as well as the sulfate content and N- to O-sulfation ratio. Fourier transform infrared (FTIR) spectra indicated that GAG extract presented similar structural characteristics to bovine heparan sulfate and chondroitin sulfate. The pattern of extract migration in agarose gel electrophoresis and specific digestion with chondroitinase ABC and heparinase III indicated the presence of a mixture of chondroitin sulfate-type GAG, as main component, and heparan sulfate-type GAG. Free radical scavenging and ferric ion reducing assays showed that GAG extract had high antioxidant activity, which slightly decreased after enzymatic treatment. *In vitro* MTT and Live/Dead assays showed that GAG extract had the ability to inhibit cell proliferation in human Hep-2 cell cultures, at cytocompatible concentrations in normal NCTC clone L929 fibroblasts. This capacity decreased after enzymatic digestion, in accordance to the antioxidant activity of the products. Tumoral cell migration was also inhibited by GAG extract and its digestion products. Overall, GAG extract from *R*. *venosa* marine snail exhibited antioxidant and antiproliferative activities, suggesting its potential use as novel bioactive compound for biomedical applications.

## 1. Introduction

Glycosaminoglycans (GAG) are anionic mucopolysaccharides with a long, linear chain of repeating disaccharide units, consisting of a hexosamine and an uronic acid. GAG vary as molecular weight, sulfation pattern and degree, and core structure, comprising four major groups: chondroitin sulfate (CS)/dermatan sulfate (DS), heparin/heparan sulfate (HS), keratan sulfate and hyaluronan/hyaluronic acid [1, 2]. Thus, CS and HS are sulfated GAGs, while hyaluronic acid is a non-sulfated GAG. Usually, GAG are covalently bound to a core protein, forming proteoglycans located in the extracellular matrix of the connective tissue of vertebrates and invertebrates, and are also found in bacteria.

In recent years, GAG isolated from marine organisms were investigated as compounds presenting a wide range of biological activities, including antioxidant [3, 4], antimicrobial [3], antiproliferative [5, 6], immunomodulatory [7], hypolipidemic [8], anticoagulant and antithrombin activity [9–11]. Among marine GAGs, CS and HS were studied as supplements for arthritis and thromboembolic disease treatment [12].

Marine invertebrates are usually consumed as seafood due to their nutritional value. Several marine species have been reported to contain GAG with various biological activities. Thus, GAG-type polysaccharidic extracts with anticoagulant properties were isolated from *Haliotis discus hannai* Ino gastropod [11] and from *Anodonta anodonta*, *Nodipecten nodosus*, and *Donax cuneatus* mollusks [13–15]. In a recent study, GAG with CS chains from *Perna canaliculus* green lipped-mussel exhibited neurite outgrowth activity [16], while *Lapemis curtus* sea snake polysaccharides fractions containing CS and DS proved to have scavenging ability of 2,2-diphenyl-1-picrylhydrazyl (DPPH) radicals, iron chelating properties and total antioxidant capacity [4]. Also, sulfated polysaccharides with a dermatan structure were isolated from the starfish *Lethasterias fusca* and were investigated as stimulators of hematopoiesis [17]. According to the developmental stage, tissue used for extraction and harvest season, different GAG types were identified, such as glucosan analogues found in some mussels and clams, and GAG and proteoglycans specific for octopuses and snails [12].

*Rapana venosa* is a predatory marine snail belonging to the Muricidae family, native to the Western Pacific and accidentally introduced into the Black Sea. *R*. *venosa* consumes different species of mussels and molluscs, causing ecosystem changes [18]. This marine invertebrate is mainly exploited in the food industry, but it can also be used as a bioindicator to monitor the accumulation of different pollutants [19, 20]. In addition, the soft tissue of *R*. *venosa* was valorized as a rich source of bioactive compounds, such as amino acids, lipids and proteins, which demonstrated wound healing properties and antioxidant activity [21–23]. A recent study has reported that gelatin isolated from *R*. *venosa* marine snail could represent an alternative to that from terrestrial vertebrates [24]. Previous studies have provided information on a heparin-type of GAG isolated from *R*. *venosa*, known as raparin [25, 26], but only scarce information regarding its chemical characteristics were provided. No studies were found on *R*. *venosa* GAG extract and its enzymatic digestion products concerning their redox properties and antiproliferative effect in squamous cell carcinoma model.

The aim of the present study was to isolate a GAG extract from the soft tissue of *R*. *venosa* marine snail and to investigate its physicochemical, antioxidant and antiproliferative properties for further valorization in biomedical products. Its free radicals scavenging and ferric reducing antioxidant power, as well as its antiproliferative effect in experimental models of squamous cell carcinoma were comparatively assessed to its enzymatic digestion products.

## 2. Materials and methods

### 2.1. Materials

Biological material consisted of specimens of the marine snail *R*. *venosa* collected in August 2018 from the Romanian Black Sea coast between 2 Mai and Vama Veche areas. They were identified at the University "Ovidius" of Constanta and stored at -20°C.

Chondroitin 4-sulfate sodium salt (CS) from bovine trachea, heparan sulfate sodium salt (HS) from bovine intestinal mucosa, chondroitinase ABC (Chase ABC) from *Proteus vulgaris* (E.C. 4.2.2.20) and heparinase III (Hep III) from *Flavobacterium heparinum* (E.C. 4.2.2.8) were purchased from Sigma Aldrich (Germany). NCTC clone L929 murine fibroblasts and human Hep-2 squamous carcinoma cells were obtained from the European Collection of Authenticated Cell Cultures (ECACC). Minimum essential medium (MEM), fetal bovine serum (FBS), 3-(4,5 dimethylthiazol2-yl)-2,5 diphenyltetrazolium bromide (MTT) and 2,2’-azino-bis(3-ethyl-benzo-thiazoline-6-sulfonic acid) diammonium salt (ABTS) were acquired from Sigma-Aldrich (Germany). All other chemicals were of analytical purity and purchased from Sigma-Aldrich (Germany), unless otherwise specified.

### 2.2. Preparation of *R*. *venosa* GAG extract

For GAG extraction, the soft tissue was cut into 1 cm^3^ pieces and defatted by incubation in acetone, in a ratio of 1:4 (w/v), for 24 h. After thorough washing in distilled water, a total extract was obtained by alkaline treatment in 0.5 M NaOH solution, in a ratio of 1:10 (w/v) under gentle stirring, overnight. The extract was centrifuged at 2000g, for 20 min and 1% celite was added to the supernatant, in order to eliminate proteins. The resulting clear solution was treated with 3 volumes of cold ethanol containing 0.5% sodium acetate, at 4°C, for 24 h and then, centrifuged at 7000g, for 30 min. The precipitate representing purified GAG extract was dissolved in distilled water and lyophilized at a freeze-dryer equipment (Martin-Christ, Germany). The yield was calculated as percentage from initial mass on a dry matter basis.

### 2.3. Chemical analysis of GAG extract

Total carbohydrates content was determined by anthrone method, using a standard curve of glucose in the range of concentrations 0–100 μg/mL [27]. The hexosamine content was determined using the Ehrlich reagent method [28]. A standard curve was built using glucosamine hydrochloride in the range of concentrations 0.03–0.15 mM. Determination of uronic acids content was performed using the orcinol-based method, as previously described [29]. The standard curve was built using glucuronic acid in the range of concentrations 0.03–0.15 mM. Total protein content was determined using the Biuret method and bovine serum albumin as a standard in the range of concentrations 0–10 mg/mL. Sulfate content was estimated by barium chloride assay [30], using potassium sulfate in the range of 0.25–4 mg/mL to build the standard curve. All spectrophotometric analyses were carried out at a V-650 UV-VIS spectrophotometer (Jasco, Japan). The content of *O-* and *N-*sulfated GAGs and their ratio were determined using Blyscan sulfated GAG assay kit (Biocolor, UK), according to the manufacturer’s instructions. CS sodium salt from bovine trachea was used to build the standard curve. The absorbance was read at a SPECTROstar nano microplate reader (BMG Labtech, Germany). A number of six measurements was taken for each chemical analysis.

### 2.4. Elemental analysis by scanning electron microscopy—energy dispersive X-ray (SEM-EDX)

Elemental composition of GAG extract was estimated at a SEM equipment (Hitachi SEM SU1510, UK) fitted with an EDX spectrometer (Oxford Instruments, UK). Morphological information was obtained at an accelerating voltage of 5 kV and EDX measurements were taken at a live time of 120 sec. A number of ten EDX measurements was taken.

### 2.5. Fourier transform infrared (FTIR) spectroscopy

A samples of GAG extract was mixed with KBr and compressed to a transparent disk. Standards of bovine CS and HS were analyzed in similar conditions. FTIR spectra were recorded from 4000 to 500 cm^-1^ at a Bruker Tensor 27 infrared spectrometer (SpectraLab, Canada). Spectral data was analyzed using ORIGIN 8.0 software.

### 2.6. Enzymatic digestion of GAG extract

Enzymatic digestion of GAG extract was performed using two specific lyases, as previously described [5], with minor modifications. Briefly, a sample of GAG extract (1 mg) was incubated in 0.5 mL solution of 0.01 U heparinase III and 0.01 U chondroitinase ABC, respectively, supplemented with 10 mM calcium acetate and 50 mM sodium acetate buffer, pH 7.0, at 37°C, for 24 h. The enzymes were inactivated by heating to 100°C, for 5 min and the absorbance was measured at 232 nm, at a SPECTROstar nano microplate reader (BMG Labtech, Germany).

### 2.7. Agarose gel electrophoresis

Samples of GAG extract and its enzymatic digestion products (0.5 mg/mL) were migrated in 0.5% agarose gel in 0.05 M 1,2-diaminopropane/acetic acid buffer, pH 9.0, at 60 mA, for 2 h at a Biometra gel unit (Analytik Jena, Germany) [31]. Standards of bovine CS and HS were migrated in similar conditions. After migration, the samples were stained using 0.2% toluidine blue solution in a mixture of ethanol:water:acetic acid in a ratio of 50:49:1 (v/v/v), for 15 min and discolored in the same solvent mixture.

### 2.8. Evaluation of antioxidant activity

The free ABTS radical cation scavenging capacity of *R*. *venosa* GAG extract and its enzymatic digestion products was determined as previously described [32]. An ABTS stock solution was prepared by mixing 7 mM ABTS solution with 2.45 mM potassium persulfate, in a ratio of 1:1 (v/v) and incubation in the dark, at room temperature, for 16 h. For experiments, the stock solution was diluted to obtain the ABTS reagent (blank) with an absorbance (Abs) value of 0.70 ± 0.02 at 734 nm. A volume (100 μL) of different concentrations of sample between 1–5 mg/mL was added to 1 mL of ABTS reagent and incubated in the dark, at room temperature, for 6 min. The absorbance of the reaction mixtures was measured at 734 nm at a V650 UV/VIS spectrophotometer (Jasco, Japan). Trolox served as positive control and the results were expressed as Trolox equivalents (TE).

The ferric ion reducing antioxidant power (FRAP) of *R*. *venosa* GAG extract and its enzymatic digestion products was assessed as previously described [33]. FRAP reagent was prepared as a mixture of 0.1 M acetate buffer, pH 3.6, 10 mM 2,4,6-tris(2-pyridyl)-s-triazine (TPTZ) in 40 mM HCl and 20 mM ferric chloride, in a ratio of 10:1:1 (v*/*v*/*v). A volume of 2 mL FRAP reagent was added to 100 μL of sample and the mixture was incubated at 37°C, for 30 min. The absorbance was read at 593 nm at a V650 UV/VIS spectrophotometer (Jasco, Japan). A standard curve was built using iron (II) sulfate solution (0–1 mM). A number of six measurements was taken for each assay.

### 2.9. Evaluation of *in vitro* cytocompatibility

The *in vitro* cytocompatibility of GAG extract and its enzymatic digestion products was evaluated in NCTC clone L929 murine fibroblasts by direct contact method. Cells were seeded in 96-well culture plates at a density of 5x10^4^ cells/mL in MEM supplemented with 10% FBS and 1% antibiotic mixture of penicillin, steptomycin and neomycin, and incubated overnight in standard conditions of humidified atmosphere with 5% CO_2_, at 37°C, to allow cell attachment. All samples were sterile filtered through 0.2 μm syringe filters. Then, the culture medium was replaced with fresh medium containing different concentrations of sample in the range of 0.05–1 mg/mL and the plates were further incubated for 72 h. Untreated cells served as negative control, while cells treated with 100 μM H_2_O_2_ served as positive control. At the end of incubation, the cell viability was assessed using the MTT assay, which evaluates the activity of mitochondrial dehydrogenases [34]. Briefly, the culture medium was discarded and cells were incubated with MTT solution (0.25 mg/mL), at 37°C, for 3 h. The insoluble formazan crystals were dissolved in isopropanol by gentle stirring, at room temperature, for 15 min. The absorbance was recorded at 570 nm at a SPECTROstar nano microplate reader (BMG Labtech, Germany). The values were directly correlated to the number of viable cells. The results were reported as percentage from negative control, considered 100% viable. Two experiments were run in triplicate.

### 2.10. Evaluation of antiproliferative activity

The antiproliferative activity of GAG extract and its enzymatic digestion products was evaluated in human Hep-2 squamous carcinoma cell line by cell proliferation, morphology and live/dead cell ratio, and migration assays.

For *cell proliferation and live/dead* evaluation, a similar experimental model to that described for cytocompatibility testing was used. Untreated cells served as negative control, while cells treated with 60 μM curcumin served as positive control. After 72 h of incubation, cell proliferation was assessed using the MTT assay, as described above. The sample concentration that inhibited cell proliferation by 50% (IC_50_) was calculated by regression analysis. Two experiments were run in triplicate.

Cell morphology and live/dead cells ratio were assessed by fluorescence microscopy using Live/Dead viability/cytotoxicity assay kit (Molecular Probes, Thermo Fisher Scientific, USA), according to the manufacturer’s instructions. Briefly, after 72 h of incubation, cells were washed with PBS and stained with 2 μM calcein-AM and 4 μM ethidium homodimer-1, at room temperature, for 30 min. Micrographs were acquired at an Axio Observer D1 microscope provided with camera and AxioVision 4.6 software (Carl Zeiss, Germany). Live/dead cells were quantitatively determined using ImageJ 1.51 software. The experiment was run in triplicate.

For *cell migration* evaluation, an experimental model of *in vitro* scratch was used, as previously described [35]. Briefly, Hep-2 cells were seeded in 24-well culture plates at a cell density of 2x10^5^ cells/mL and incubated in standard conditions until confluence, when a linear scratch was created with a sterile 200 μl tip. Cells were washed with PBS and further incubated with different concentrations of sample (0.5 mg/mL and 1 mg/mL), for 24 h. Micrographs were acquired at an Axio Observer D1 microscope (Carl Zeiss, Germany) at the beginning of the experiment (t = 0) and after 24 h of incubation. The rate of cell migration was quantitatively determined as percentage from negative control using ImageJ 1.51 software. The experiment was run in triplicate.

### 2.11. Statistical analysis

The results were expressed as mean ± standard deviation (SD). Statistical analysis of control-sample pairs of interest was carried out using two-tailed paired Student’s *t*-test. Statistically significant differences were considered at p<0.05.

## 3. Results and discussion

### 3.1. Isolation and physicochemical characterization of GAG extract

A GAG extract was isolated from the soft tissue of *R*. *venosa* marine snail after defatting, deproteinization and ethanol precipitation, with a yield of 42.42±3.65 mg/g dry weight (d.w.).

**Fig 1:**
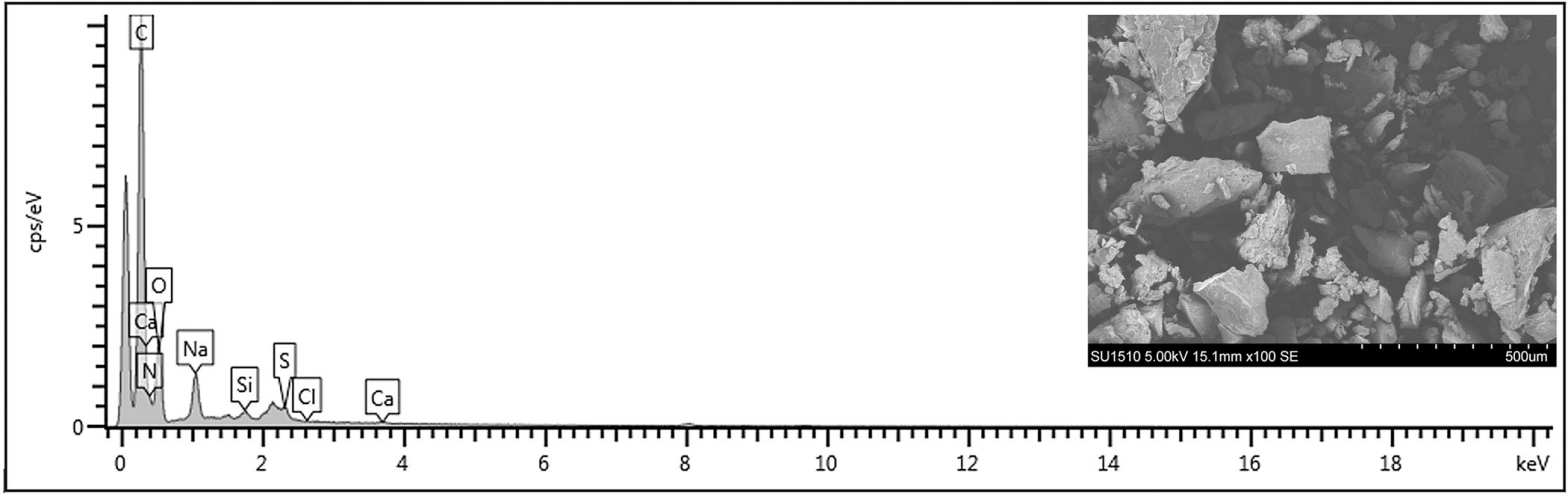
EDX spectra of GAG extract isolated from *Rapana venosa* marine snail.

SEM observations on GAG extract showed the presence of particles with irregular shapes and a rough surface (Fig 1). The elemental composition of the extract, determined by EDX spectral analysis (Fig 1) revealed the presence of carbon, oxygen, nitrogen, sodium and sulfur (Table 1). Traces of silica and calcium were also detected. The same main elements were found in the extract of sulfated polysaccharides from the marine gastropod *Bursatella leachii* [36]. The nitrogen content might indicate the presence of hexosamines, while the sulfur content corresponds to the presence of sulfate groups, representing specific structural features of GAGs [36].

**Table 1:** The chemical and elemental composition of GAG extract from *Rapana venosa* marine snail. The results were expressed as mean ± SD (n = 6 for chemical composition and n = 10 for elemental composition).

Chemical composition	Total carbohydrates (%)	Hexosamines (%)	Uronic acids (%)	Sulfate (%)	Protein (%)		
GAG extract	68.49 ± 3.01	22.64 ± 3.75	20.70 ± 2.02	13.21 ± 0.23	10.93 ± 0.38		
**Elemental composition**	**Carbon** **(%)**	**Nitrogen** **(%)**	**Oxygen** **(%)**	**Sodium** **(%)**	**Sulfur** **(%)**	**Silica** **(%)**	**Calcium** **(%)**
GAG extract	60.41 ± 4.31	14.33 ± 2.85	21.94 ± 3.63	1.93 ± 0.62	0.77 ± 0.23	0.38 ± 0.02	0.13 ± 0.01

The chemical composition of marine GAG extract is shown in Table 1. The extract contained high amount of carbohydrates (68.49%), while the protein content was 10.93%. Hexosamines (22.64%) and uronic acids (20.70%) were quantified as the main constituents of the repetitive disaccharide units present in GAG. The sulfate content of GAG extract was found to be 13.21%, indicating the presence of sulfated derivatives. The GAG sulfation pattern varies and can occur at various positions of their chain to generate N- or O-sulfation derivatives that can modulate the biological processes through extracellular signals and cell-matrix interactions [37]. In the present study, the data showed that *N*-sulfated derivatives represented a more abundant component than *O*-sulfated derivatives and their weight ratio was 7:3. These results were in accordance to previous studies on marine invertebrates, such as the Malaysian *Stichopus vastus* and *Stichopus hermmani* sea cucumbers [38], *Carcinus maenas* shore crab [39] and *Acanthaster planci* starfish [40] presenting similar prevalence of *N*-sulfated over *O*-sulfated derivatives in GAG extract. No similar studies were found on *R*. *venosa* GAG extract.

**Fig 2:**
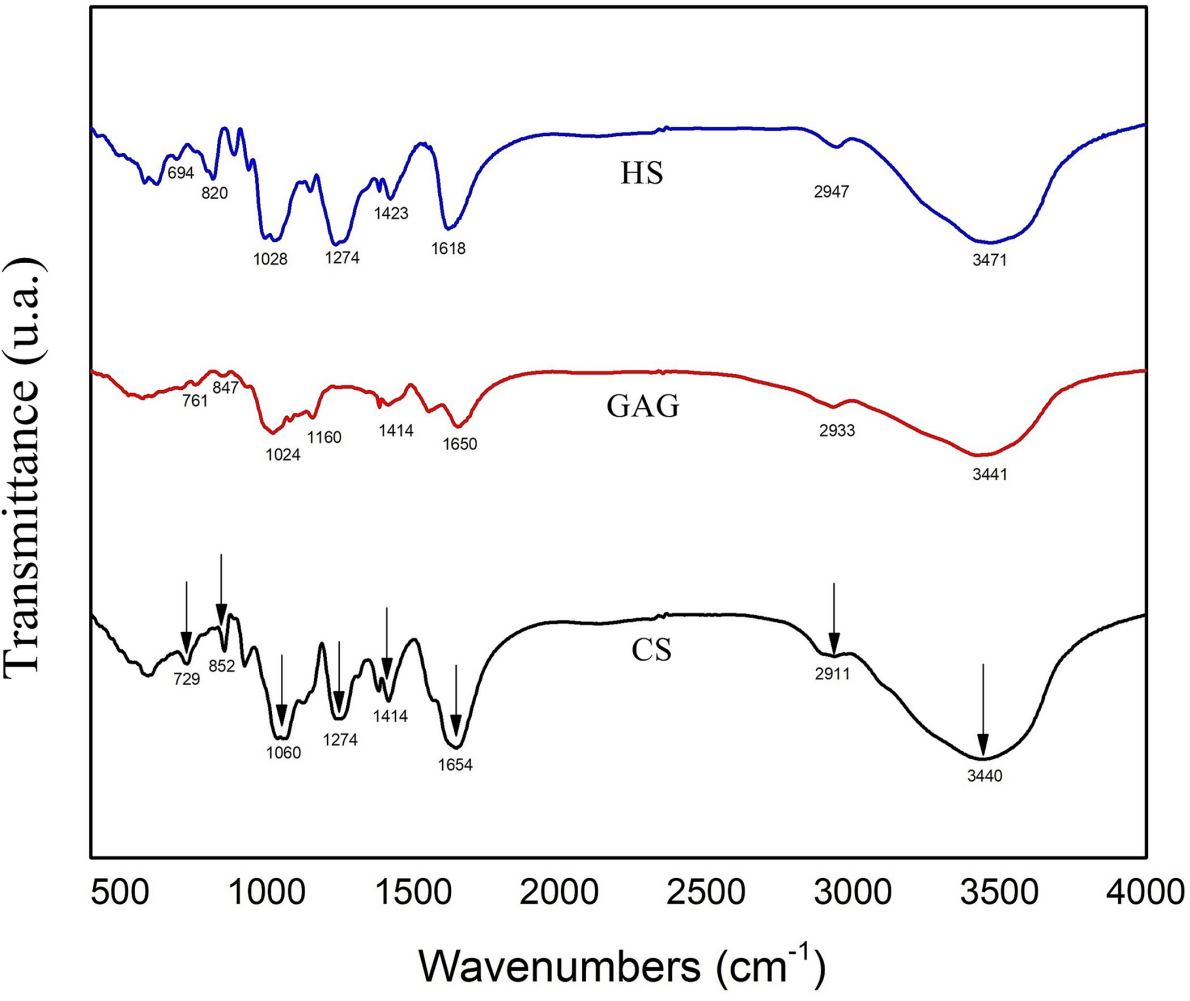
FTIR spectra of GAG extracted from *Rapana venosa* marine snail, bovine condroitin sulfate (CS) and heparan sulfate (HS).

FTIR spectrum of marine GAG extract was comparatively analysed to spectra of standards of bovine CS and HS (Fig 2). The absorption bands at 847, 852 and 820 cm^-1^ observed in the spectra of marine GAG extract, CS and HS, respectively, were attributed to the vibrational mode of C-O-S bonds. The band observed in 1024–1060 cm^-1^ region, related to C-O-C skeletal vibration, was present in all samples. The band at 1274 cm^-1^ was ascribed to the stretching vibration of S = O, in case of bovine CS and HS. However, this band was absent in the case of *R*. *venosa* GAG extract, suggesting that the degree of sulfation might vary on the molecule chain, depending on the tissue source [1, 41]. The stretching vibrations of carboxyl groups were present in 1618–1654 cm^-1^ region, while the bands from 2911, 2933 and 2947 cm^-1^ were attributed to C-H stretching vibration of the sugar ring. Also, it was noticed a broad band in the range of 3420–3470 cm^-1^, charactacteristic to O-H stretching vibration in all analyzed samples.

FTIR data indicated that the GAG extract from *R*. *venosa* marine snail presented the main structural characteristics of bovine CS and HS. Thus, the C-O-S vibration was observed in all FTIR spectra, showing the presence of both carboxyl and sulfate groups. However, marine GAG extract presented a lower sulfation degree, compared to that of vertebrates. Similar studies reported the presence of bands corresponding to C-O-S bonds and carboxyl groups in the extract of GAGs-like sulfated polysaccharides isolated from the marine gastropod *Haliotis discus* [11]. No similar studies were found on *R*. *venosa* GAG extract.

**Fig 3:**
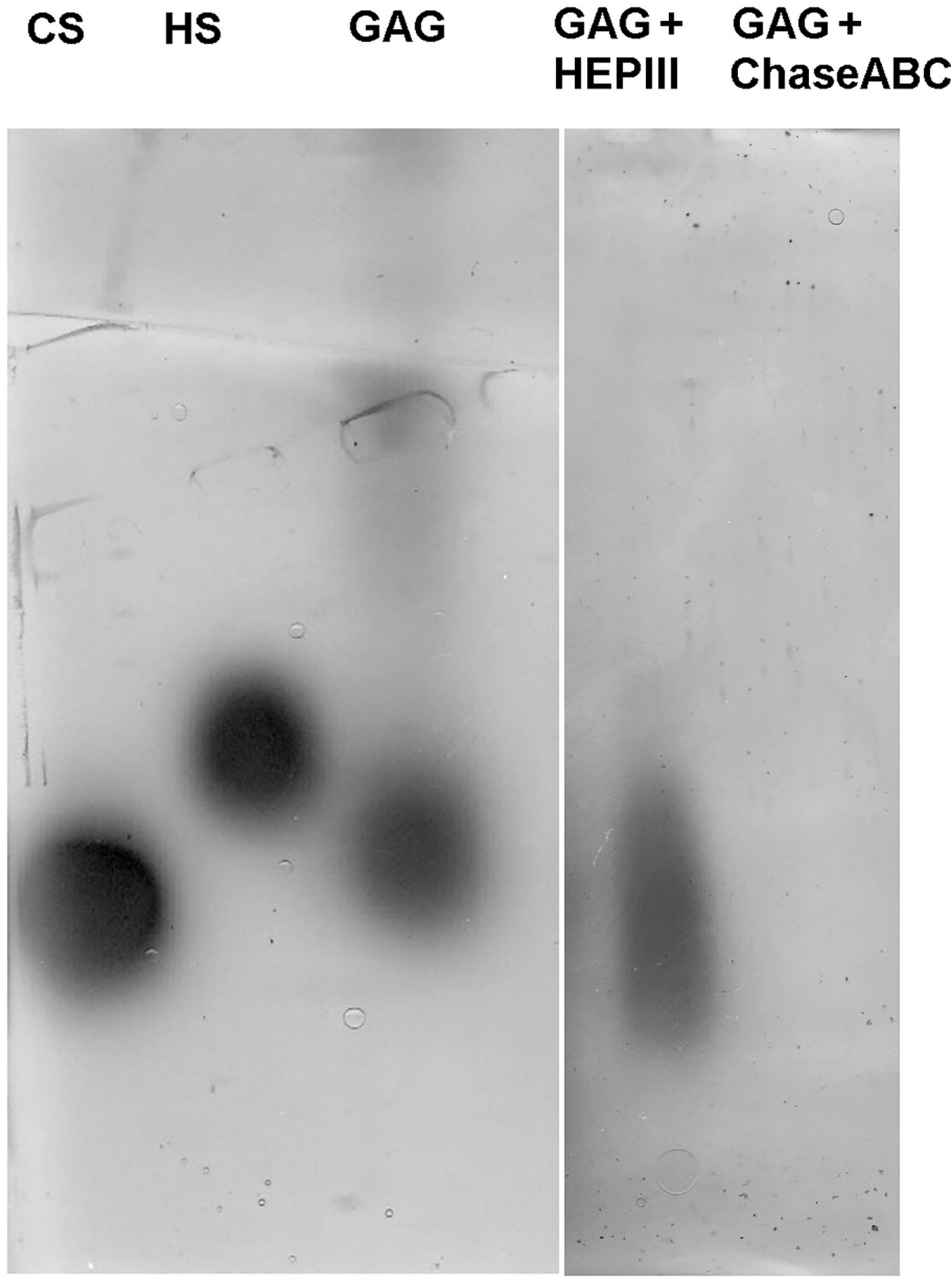
Agarose gel electrophoresis of GAG extract from *Rapana venosa* marine snail before and after incubation with heparinase III (Hep III) and chondroitinase ABC (Chase ABC). Standards of bovine condroitin sulfate (CS) and heparan sulfate (HS) migrated in similar conditions.

The electrophoretic migration pattern of marine GAG extract is shown in Fig 3 (and S1 and S2 Figs). The marine GAG extract migrated as a single metachromatic band, presenting lower mobility than bovine CS, but higher than HS. After the enzymatic treatment of GAG extract with chondroitinase ABC, no band was visible. In turn, the extract incubated with heparinase III showed a large coloured band.

GAG-degrading enzymes (lyases) can cleave specific glycosidic linkages between a hexosamine and an uronic acid, resulting in a mixture of disaccharides, tetrasaccharides and oligosaccharides that could exhibit different biological functions, compared to the long polysaccharidic chain [42]. Thus, Chase ABC cleaves sulfated and non-sulfated types of CS, while Hep III cleaves heparin and HS. Taking this into consideration, the electrophoretic data indicated that the GAG extract contained a significant amount of CS-type compounds digested by Chase ABC, resulting in oligo- and di-saccharides with lower molecular weight and nondetectable amounts of HS. Similar, Hep III digested HS-type compounds were present in the extract, leaving the CS-type compounds visualized in the electrophoregram.

The results were in agreement with previous studies reporting the electrophoretic migration of GAG extract from different mollusc species as a single band [9, 14, 15]. In addition, previous studies have reported the identification of HS-like and traces of CS-type GAG in a crude extract of *Nodipecten nodosus* mollusk, after treatment with proteolytic enzymes [14]. Similar, the main components identified in GAG isolated from *Anodonta anodonta* mollusc by digestion with specific lyases were sulfated and non-sulfated CS and heparin [13]. No similar studies were found on *R*. *venosa* GAG extract.

### 3.2. Antioxidant activity

The antioxidant activity of *R*. *venosa* GAG extract and the influence of the enzymatic degradation of the extract on this property were analysed. The results of the antioxidant activity exerted *in vitro* by *R*. *venosa* GAG extract are presented in Table 2. Data showed its capacity to scavenge free ABTS radicals (47.05 mM TE/g d.w.) and to reduce ferric ions at an extent of 10.25 mM Fe (II)/g d.w. The heparinase III digestion product exhibited lower free ABTS radical scavenging capacity (15.09 mM TE/g d.w.) and similar FRAP (9.66 mM Fe (II)/g d.w.) values to those of *R*. *venosa* GAG extract. The chondroitinase ABC digestion product had also less antioxidant capacity than the initial GAG extract, as free ABTS scavenging capacity (27.03 mM TE/g d.w.) and FRAP (6.08 mM Fe (II)/g d.w.).

**Table 2:** Antioxidant activity determined as free ABTS radicals scavenging capacity and FRAP of *Rapana venosa* GAG extract, before and after digestion with heparinase III (Hep III) and chondroitinase ABC (Chase ABC). The results were expressed as mean ± SD (n = 6).

Sample	Free ABTS radical scavenging capacity (mM TE/g d.w.)	FRAP (mM Fe (II)/g d.w.)
*R*. *venosa* GAG extract	47.05 ± 3.27	10.25 ± 0.36
GAG extract + Hep III	15.09 ± 1.56	9.66 ± 0.19
GAG extract + Chase ABC	27.03 ± 2.28	6.08 ± 0.27

Data showed that *R*. *venosa* GAG extract and the products resulted after enzymatic digestion presented antioxidant capacity. Lower values observed for the digested products could be correlated with lower molecular weight compounds, as evidentiated by electrophoresis analysis. In addition, the GAGs extract had different composition, degree of polymerization and chain structure from those of the digested products. The observed differences between the digestion products could be due to different action of the specific lyases used to degrade the GAG extract.

### 3.3. In vitro cell culture evaluation

**Fig 4:**
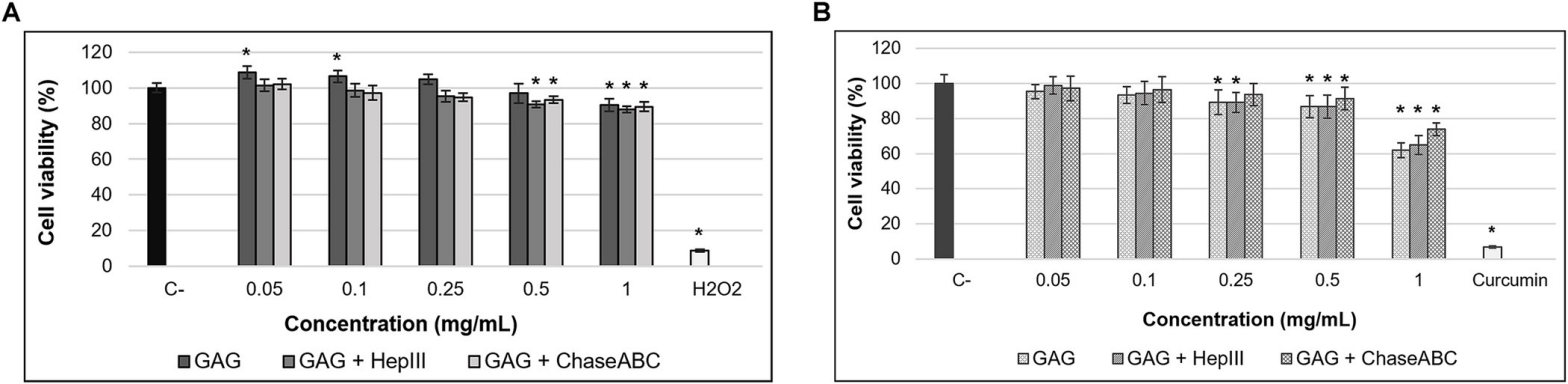
Cell viability of normal L929 cells (A) and tumor Hep-2 (B) cells after cultivation with different concentrations of marine GAG extract from *Rapana venosa*, before and after enzymatic digestion with heparinase III (Hep III) and chondroitinase ABC (Chase ABC) for 72 h, assessed by MTT assay. The results are expressed as mean values ± SD (n = 6). *p<0.05, compared to negative control (C-).

*In vitro* cytocompatibility of marine GAG extract and its enzymatic digestion products was assessed in normal fibroblast-like L929 cells. As shown in Fig 4A, all samples showed values of cell viability above 80% (non-cytotoxic effect), at each tested concentration between 0.05–1 mg/mL, indicating good cytocompatibility. Thus, the cell viability ranged between 90.37–108.74% for GAG extract, between 87.90–101.53% for heparinase III digestion product and between 89.57–102.20% for chondroitinase ABC digestion product. In case of cells treated with 0.05 mg/mL and 0.1 mg/mL marine GAG extract, cell viability values (107.92% and 106.51%, respectively) were significantly (p<0.05) higher than that of negative control (100%), showing the capacity to stimulate the proliferation of normal cells. All cytocompatible concentrations were further tested for antiproliferative activity.

The antiproliferative activity of marine GAG extract and its enzymatic digestion products was assessed as their inhibitory effect on tumor cell proliferation, morphology and percentage of live/dead cells and migration in a model of Hep-2 squamous cell carcinoma. Based on MTT results, a concentration of 1 mg/mL marine GAG extract significantly (p<0.05) decreased Hep-2 proliferation by 37.99% at 72 h post-treatment (Fig 4B). The inhibition was also exerted by a concentration of 1 mg/mL digestion product of heparinase III by 35.01% and that of chondroitinase ABC by 26.10%. The IC_50_ values were calculated at 1.42 mg/mL for *R*. *venosa* GAG extract, 1.5 mg/mL for extract incubated with heparinase III and 2.06 mg/mL for chondroitinase ABC digestion product. Taking into account that the concentration of 1 mg/mL was cytocompatible in normal cell culture, these results demonstrated an antiproliferative effect of *R*. *venosa* GAG extract and its digestion products against Hep-2 cells.

These findings were in line with previous studies on GAG-like polysaccharidic extracts from different marine invertebrates, such as algae, ascidians and molluscs with antiproliferative activity in various cancer cell lines [5, 6, 43, 44]. Thus, potential antitumor properties were described for disaccharides (carrabioses) isolated from red seaweed in murine mammary adenocarcinoma LM2 cells [35]. Aldairi et al. [5] have reported the antiproliferative activity of marine GAG-like polysaccharides from the common cockle (*Cerastoderma edule*) and its digestion fragments obtained after enzymatic treatment with heparinases and chondroitinase ABC in leukaemia cell lines (K562 and MOLT4). They have suggested that the HS/heparin-like GAG were responsible for this activity, while the CS-like components contributed to a lesser extent to the antitumor effect. Significant inhibitory activity of breast cancer cell proliferation was reported for the GAG extract isolated from the common whelk (*Buccinum undatum*) and the fragments obtained after enzymatic digestion with heparinases and chondroitinase ABC [6]. However, the antiproliferative activity of the fragments generated by heparinases was reduced, in comparison to those generated by chondroitinase ABC. It was previously found that the antiproliferative activity of marine GAG extract from *Sanguinolaria acuta* mollusk was directly correlated to its free radicals scavenging activity [45]. Similarly, *R*. *venosa* GAG extract from the present study has exhibited the strongest antioxidant activity *in vitro* and has also displayed the best inhibitory effect on Hep-2 cell proliferation.

**Fig 5:**
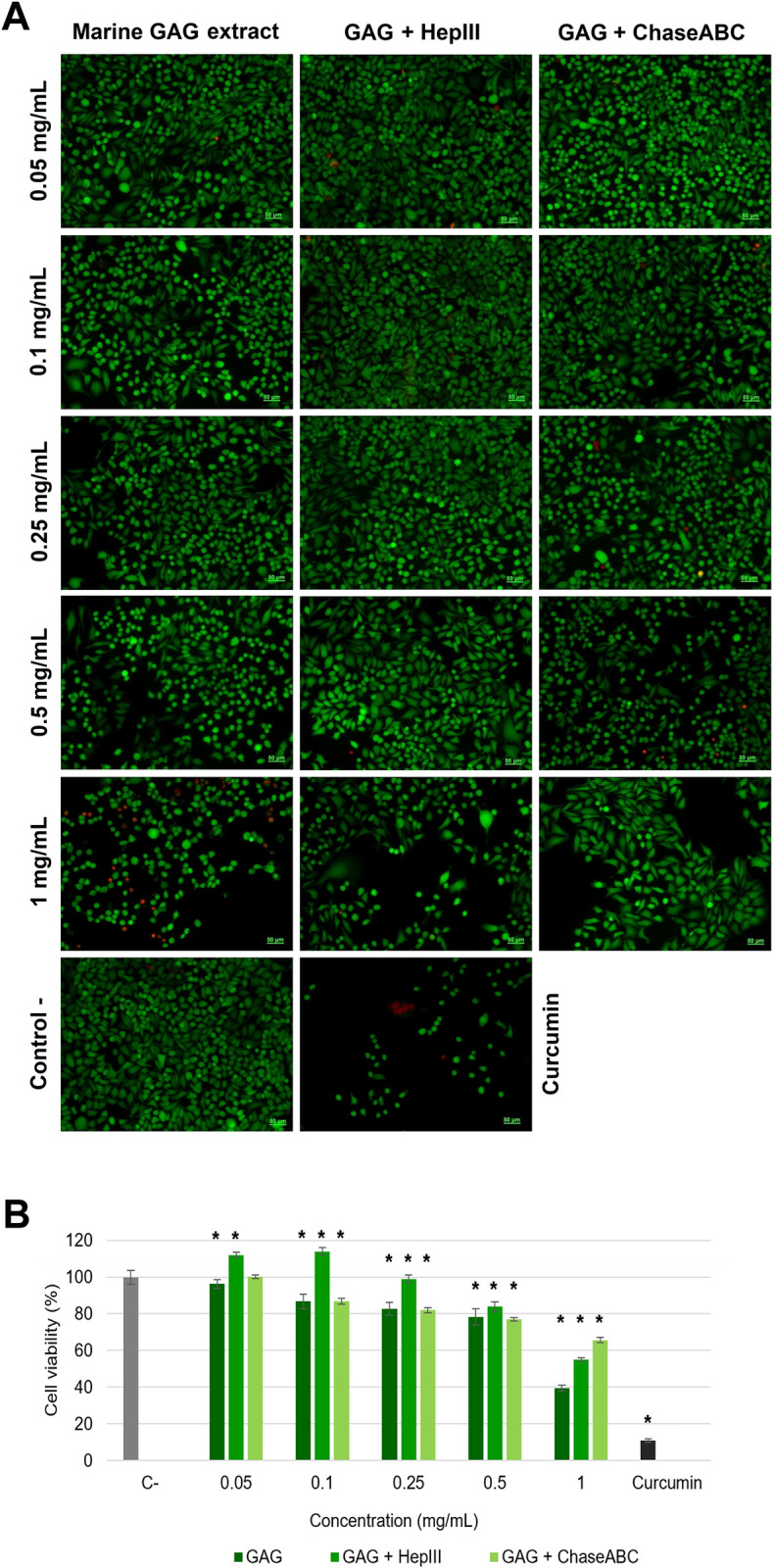
Micrographs of live (green) and dead (red) Hep-2 cells treated with different concentrations of *Rapana venosa* GAG extract, before and after digestion with heparinase III (Hep III) and chondroitinase ABC (Chase ABC), for 72 h, assessed by Live/Dead assay (A). Scale bar = 50 μm. Histogram of cell viability determined by ImageJ analysis (n = 3) (B).

The morphology of tumor Hep-2 cells and the live/dead cells ratio after treatment with *R*. *venosa* GAG extract and its enzymatic digestion products were also analyzed. Based on Live/Dead staining, the fluorescent microscopy observations confirmed the results of MTT assay. At a concentration of 1 mg/mL, all analyzed samples induced morphological changes of Hep-2 cells, indicating that most cells became spherical (Fig 5A). The quantitative analysis showed a decrease of cell viability down to 40% for marine GAG extract, 55 and 66% for the digestion products of heparinase III and chondroitinase ABC, respectively (Fig 5B). In turn, Hep-2 cells maintained their viability higher than 77% for all samples at lower concentrations of 0.05–0.5 mg/mL and showed no significant morphological changes. They maintained the normal phenotype, similar to that of untreated cells.

**Fig 6:**
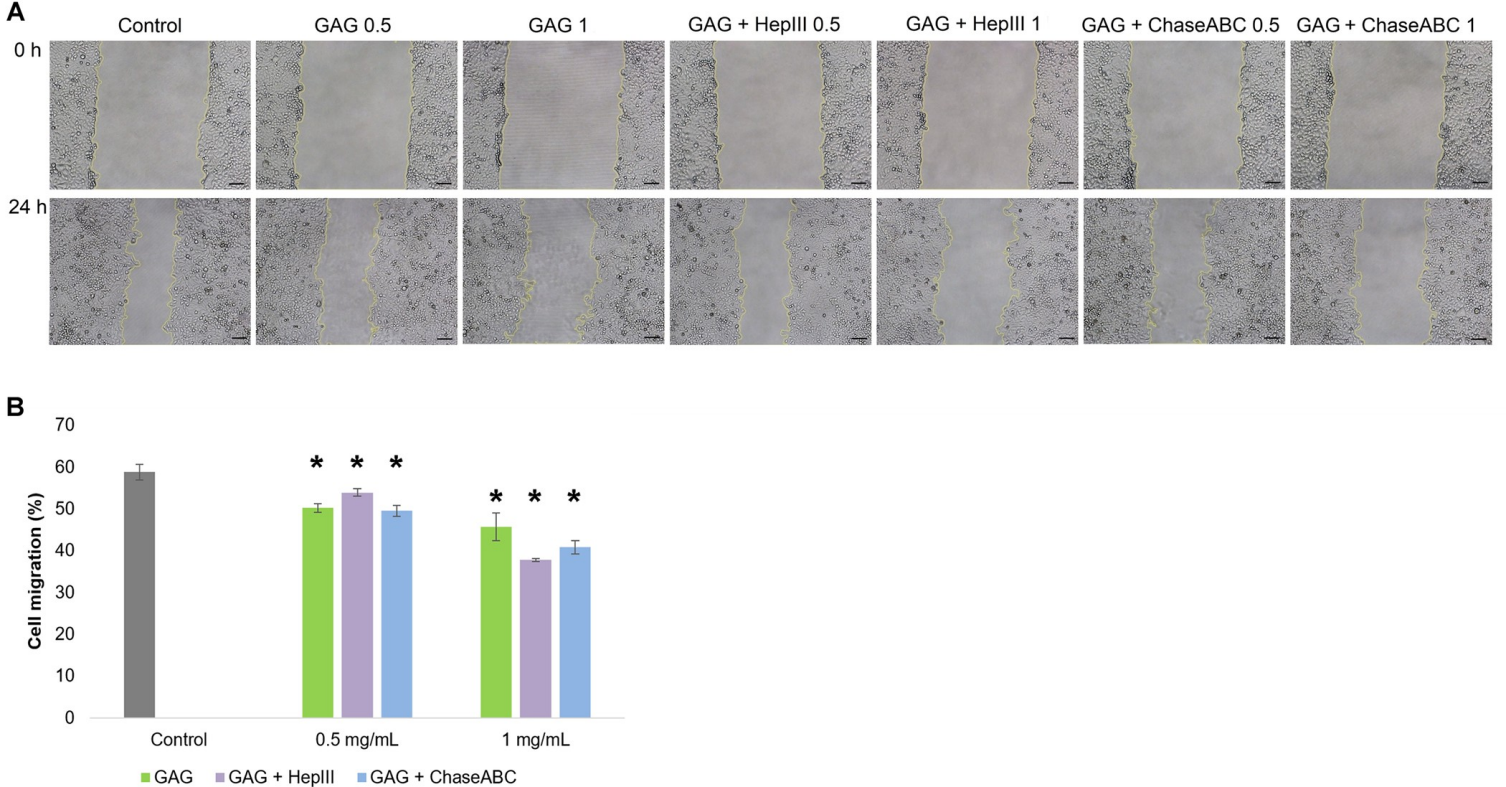
Micrographs of cell migration of Hep-2 scratched cells treated with *Rapana venosa* GAG extract and its digestion products with heparinase III (HepIII) and chondroitinase ABC (Chase ABC) for 24 h (A). Scale bar = 100 μm. The rate of cell migration determined by ImageJ analysis (B). The results were expressed as mean values ± SD (n = 3). *p<0.05, compared to negative control.

Based on scratch assay results, the inhibitory effect of marine GAG extract and its enzymatic digestion products on tumor Hep-2 cell migration was revealed. The acquired images showed that the migration capacity of the treated cells was reduced, compared to that of the untreated cells (Fig 6A). The quantitative analysis indicated that at a concentration of 0.5 mg/mL, cell migration decreased from 58.8% in control cells to 50.2% in GAG extract treated cells, 53.9% in cells treated with extract digested with heparinase III and 49.5% in those incubated with chondroitinase ABC digestion products (Fig 6B). At a concentration of 1 mg/mL, the percentage of cell migration decreased significantly (p<0.05), down to 45.7% for cells treated with GAG extract, 40.8% for cells treated with extract digested with chondroitinase ABC and 37.8% for cells treated with extract digested with heparinase III. All these results demonstrated that tumor cells proliferation and viability were more inhibited by marine GAG extract, while tumor cells migration was more inhibited by its digestion products. This process was well correlated to FRAP of analyzed samples, which was in accordance to a recent study showing that the agents presenting the capacity to modulate intracellular iron could represented a novel anti-cancer strategy [46]. The disruption mechanism of electron transfer to reduce iron was involved in decreasing the production of ATP, impairing F-actin cytoskeleton assembly and migration of cancer cells [47].

## 4. Conclusions

The present study demonstrated for the first time that a valuable bioactive GAG extract could be obtained by alkaline treatment of the soft tissue of the predator marine snail *R*. *venosa* and ethanol/sodium acetate purification. The extract contained *N*- and *O*-sulfated constituents in a weight ratio of 7:3 and presented the same functional groups as bovine CS and HS, but a different sulfation pattern and degree, based on FTIR spectroscopy. *R*. *venosa* GAG extract was susceptible to heparinase III and chondroitinase ABC treatments, demonstrating the presence of a mixture of CS-like and HS-like polysaccharides. Free ABTS radical scavenging and FRAP assays showed that GAG extract had antioxidant capacity, which decreased after enzymatic digestion. Cell culture studies have indicated that *R*. *venosa* GAG extract and its digestion products were cytocompatible in L929 normal cells, but had the ability to inhibit cell proliferation and viability of Hep-2 squamous cell carcinoma, in accordance to their antioxidant activity. Cancer cell migration was also inhibited by the digestion products in a higher degree than by marine GAG extract. All these data suggested further characterization of *R*. *venosa* GAG extract together with *in vivo* testing in order to be valorized as antioxidant and antiproliferative agent.

## Supporting information

S1 Fig Agarose gel electrophoresis of GAG extract from marine snail *Rapana venosa* before incubation with heparinase III (Hep III) and chondroitinase ABC (Chase ABC). Standards of bovine condroitin sulfate (CS) and heparan sulfate (HS) migrated in similar conditions. 1 –CS; 2 –HS; 3 –GAG; 4—no sample; 5 –CS; 6 –HS; 7,8,9,10—no sample.
(DOCX)

S2 Fig Agarose gel electrophoresis of GAG extract from marine snail *Rapana venosa* after incubation with heparinase III (Hep III) and chondroitinase ABC (Chase ABC). 1—plant polysaccharides; 2 - plant polysaccharides; 3 –CS; 4 –HS; 5—no sample; 6—GAG+HepIII; 7—GAG+ChaseABC; 8, 9, 10—no sample.
(DOCX)

S1 Raw data EDX analysis, cell viability, live/dead quantitative analysis, scratch assay. (XLSX)
