# Supplementary material for: Antioxidant and antiproliferative effect of a glycosaminoglycan extract from Rapana venosa marine snail
Source: PLoS One. 2024 Feb 15;19(2):e0297803. doi: 10.1371/journal.pone.0297803 (PMC10868805; doi:10.1371/journal.pone.0297803)
Supplement: S1 Fig — Standards of bovine condroitin sulfate (CS) and heparan sulfate (HS) migrated in similar conditions. 1 –CS; 2 –HS; 3 –GAG; 4—no sample; 5 –CS; 6 –HS; 7,8,9,10—no sample. (DOCX) [file pone.0297803.s001.docx]

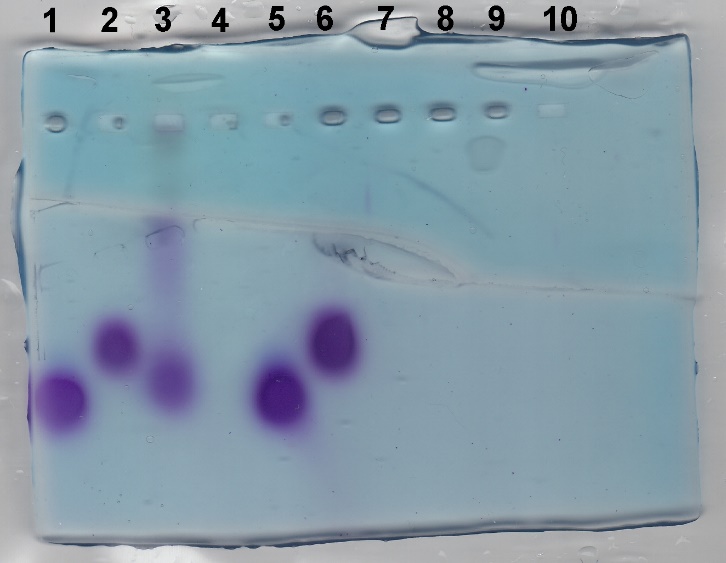


**S1 Fig.** Agarose gel electrophoresis of GAGs extract from marine snail *Rapana venosa* before incubation with heparinase III (Hep III) and chondroitinase ABC (Chase ABC). Standards of bovine condroitin sulfate (CS) and heparan sulfate (HPS) migrated in similar conditions. 1 – CS; 2 – HPS; 3 – GAGs; 4 - no sample; 5 – CS; 6 – HPS; 7,8,9,10 - no sample
