# Supplementary material for: Antioxidant and antiproliferative effect of a glycosaminoglycan extract from Rapana venosa marine snail
Source: PLoS One. 2024 Feb 15;19(2):e0297803. doi: 10.1371/journal.pone.0297803 (PMC10868805; doi:10.1371/journal.pone.0297803)
Supplement: S2 Fig — 1—plant polysaccharides; 2 - plant polysaccharides; 3 –CS; 4 –HS; 5—no sample; 6—GAG+HepIII; 7—GAG+ChaseABC; 8, 9, 10—no sample. (DOCX) [file pone.0297803.s002.docx]

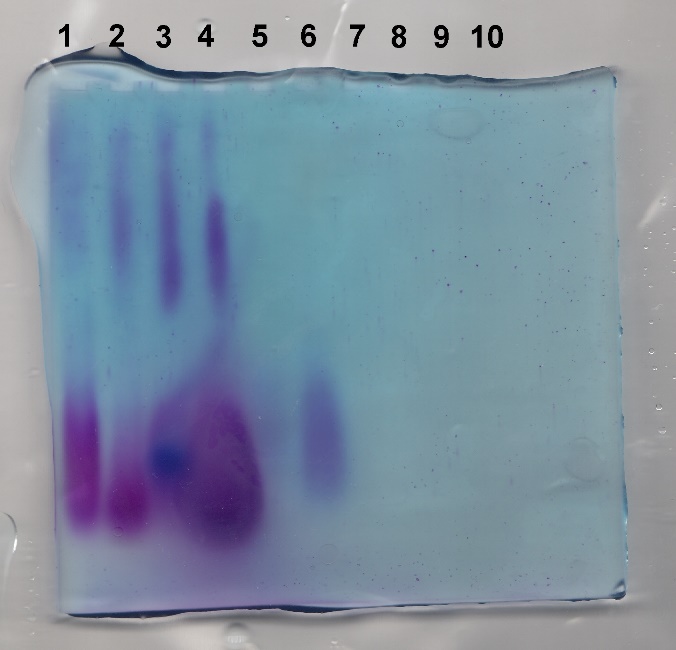


**S2 Fig.** Agarose gel electrophoresis of GAGs extract from marine snail *Rapana venosa* after incubation with heparinase III (Hep III) and chondroitinase ABC (Chase ABC). 1 - plant polysaccharides; 2 - plant polysaccharides; 3 – CS; 4 – HPS; 5 - no sample; 6 - GAGs+HepIII; 7 - GAGs+ChaseABC; 8, 9, 10 - no sample.
